# Intact regulation of G1/S transition renders esophageal squamous cell carcinoma sensitive to PI3Kα inhibitors

**DOI:** 10.1038/s41392-023-01359-x

**Published:** 2023-04-12

**Authors:** Xu Zhang, Yuxiang Wang, Xi Zhang, Yanyan Shen, Kang Yang, Qingyang Ma, Yuemei Qiao, Jiajie Shi, Yi Wang, Lan Xu, Biyu Yang, Gaoxiang Ge, Landian Hu, Xiangyin Kong, Chunhao Yang, Yi Chen, Jian Ding, Linghua Meng

**Affiliations:** 1 Division of Anti-tumor Pharmacology, State Key Laboratory of Drug Research, Shanghai Institute of Materia Medica, Chinese Academy of Sciences, Shanghai, 201203 China; 2 University of Chinese Academy of Sciences, Beijing, 100049 China; 3 School of Chinese Materia Medica, Nanjing University of Chinese Medicine, Nanjing, 210023 China; 4 Key Laboratory of Tissue Microenvironment and Tumor, Shanghai Institute of Nutrition and Health, Chinese Academy of Sciences, Shanghai, 200031 China; 5 State Key Laboratory of Cell Biology, Shanghai Institute of Biochemistry and Cell Biology, Center for Excellence in Molecular Cell Science, Chinese Academy of Sciences, Shanghai, 200031 China; 6 Department of Medicinal Chemistry, Shanghai Institute of Materia Medica, Chinese Academy of Sciences, Shanghai, China

**Keywords:** Molecular medicine, Predictive markers

## Abstract

Phosphatidylinositol 3-kinase alpha (PI3Kα) inhibitors are currently evaluated for the therapy of esophageal squamous cell carcinoma (ESCC). It is of great importance to identify potential biomarkers to predict or monitor the efficacy of PI3Kα inhibitors in an aim to improve the clinical responsive rate in ESCC. Here, ESCC PDXs with *CCND1* amplification were found to be more sensitive to CYH33, a novel PI3Kα-selective inhibitor currently in clinical trials for the treatment of advanced solid tumors including ESCC. Elevated level of cyclin D1, p21 and Rb was found in CYH33-sensitive ESCC cells compared to those in resistant cells. CYH33 significantly arrested sensitive cells but not resistant cells at G1 phase, which was associated with accumulation of p21 and suppression of Rb phosphorylation by CDK4/6 and CDK2. Hypo-phosphorylation of Rb attenuated the transcriptional activation of SKP2 by E2F1, which in turn hindered SKP2-mediated degradation of p21 and reinforced accumulation of p21. Moreover, CDK4/6 inhibitors sensitized resistant ESCC cells and PDXs to CYH33. These findings provided mechanistic rationale to evaluate PI3Kα inhibitors in ESCC patients harboring amplified *CCND1* and the combined regimen with CDK4/6 inhibitors in ESCC with proficient Rb.

## Introduction

Esophageal cancer is the seventh most common cancer and the sixth leading cause of cancer-related mortality in the world.^1^ Esophageal cancer can be divided into two major subtypes as esophageal squamous cell carcinoma (ESCC) and esophageal adenocarcinoma, which possess distinct epidemiology, pathogenesis as well as molecular profiles. ESCC represents the majority of esophageal cancer worldwide, especially in Asian and African populations.^2–4^ Currently, the therapeutic options are limited for ESCC. Endoscopic or surgical treatment is practiced in patients at early-stage, while radiotherapy or chemotherapy is predominant for advanced or metastatic ESCC.^5^ Immunotherapy with pembrolizumab, the antibody against PD-1, was recently approved for therapy of ESCC with high expression of PD-L1. The overall 5-year survival rate of ESCC patients is less than 20% up to date.^6,7^ ESCC remains a major unmet medical need worldwide.

The phosphatidylinositol 3-kinase (PI3K) integrates signals from diversified environmental cues and regulates various cellular processes including metabolism, proliferation, apoptosis and cytoskeletal rearrangement.^8^ Large scale next-generation sequencing has evidenced that the PI3K pathway is often hyper-activated in ESCC.^9^ Mutation or amplification of *PIK3CA* encoding the catalytic subunit p110α has been found in 13% or 30% of ESCC patients respectively.^10^ Moreover, hyper-activation of receptor tyrosine kinases (RTKs) or functional loss of *PTEN* also results in active PI3K signaling. Specific targeting PI3Kα has emerged as a potential approach for the therapy of ESCC. As the first PI3Kα-selective inhibitor approved by the Food and Drug Administration, alpelisib is currently tested in ESCC patients in clinical trials.^11^ Due to the high heterogeneity in the genetic alterations in ESCC, the response of ESCC to BKM120, a pan-PI3K inhibitor, was highly variable.^12^ It is of great importance to identify potential biomarkers to predict or monitor the efficacy of PI3Kα inhibitors in an aim to improve the clinical responsive rate in ESCC. *PIK3CA* mutation has been recommended as a sensitive biomarker for alpelisib for the treatment of hormone receptor (HR)-positive, human epidermal growth factor receptor 2 (HER2)-negative advanced breast cancer due to the promising results from a SOLAR-1 phase III trial (NCT02437318).^13^ As *PIK3CA* mutation is much less frequent in ESCC than that in breast cancer, it would be worthwhile to discover additional biomarkers capable of predicting the efficacy of PI3Kα inhibitors on a genome-wide scale.

CYH33 is a novel PI3Kα-selective inhibitor with a distinctive chemical structure, which was discovered in our previous work and is in phase I-II clinical trials for the therapy of advanced solid tumors including ESCC (NCT05043922, NCT04586335 and NCT03544905).^14,15^ CYH33 demonstrated a manageable safety profile, linear pharmacokinetics, and encouraging preliminary anti-tumor activity.^15–17^ Preliminary results indicated that *PIK3CA*-mutated ESCC patients are responsive to CYH33 treatment. We have reported that CYH33 exhibited potent activity to inhibit the proliferation of ESCC cells and the growth of xenografts derived from ESCC cell lines and patients (PDXs).^18,19^ Herein, we comprehensively evaluated the activity of CYH33 against 24 lines of ESCC cells and found that its activity was variable among these ESCC models. To identify biomarker potentially predicting the efficacy of CYH33, we profiled the whole genome sequencing of 14 ESCC PDXs and their sensitivity to CYH33 and alpelisib. We found that ESCC PDXs with *CCND1* amplification were more sensitive to PI3Kα inhibition. CYH33 arrested sensitive ESCC cells at G1 phase via the p21-Rb-E2F1-SKP2 positive feedback loop. CDK4/6 inhibitors sensitized resistant ESCC cells and PDXs to CYH33. These findings provided potential candidate biomarkers for PI3Kα inhibitors in treating ESCC patients and proposed rationale to improve the efficacy of PI3Kα inhibitors by simultaneously targeting CDK4/6.

## Results

### CYH33 displayed potent while variable anti-proliferative activity in ESCC cells

**Fig. 1:**
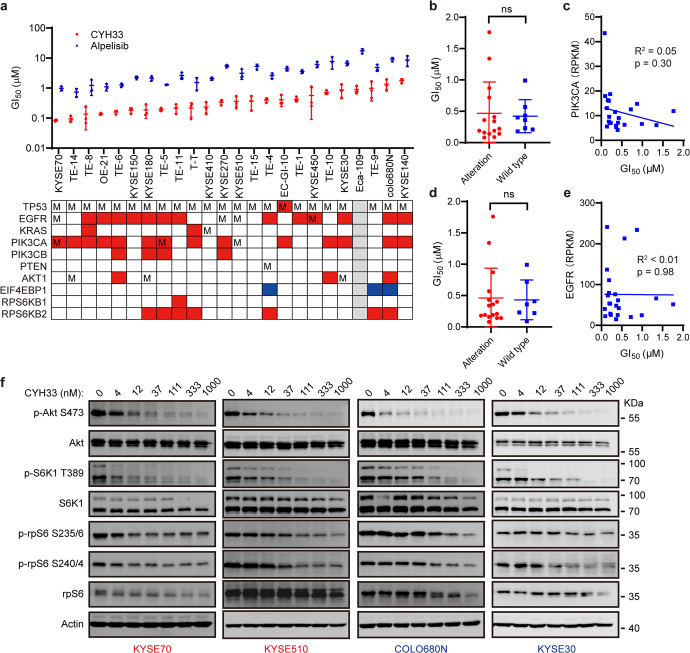
CYH33 displayed potent while variable anti-proliferative activity in ESCC cells. **a** The half maximal growth inhibitory concentration (GI_50_) values of CYH33 and alpelisib against the proliferation of 24 lines of ESCC cells. Data were presented as mean ± SD (*n* = 3). Boxes below the chart indicated the genetic alterations in ESCC cells (M, mutation; Red, amplification; Blue, copy number loss; Grey, no data). Genetic alterations were obtained from CCLE database (https://sites.broadinstitute.org/ccle/). **b**, **d** Scatter plots of mean GI_50_ values of CYH33 in cells grouped by alterations in *PIK3CA* (**b**) and *EGFR* (**d**). Difference between groups was analyzed by two-tailed unpaired Student’s *t*-test. ns, *p* > 0.05. **c**, **e** Pearson correlation analysis of the mean GI_50_ values of CYH33 and RPKM values of *PIK3CA* (**c**) or *EGFR* (**e**) was presented. RPKM values of *PIK3CA* and *EGFR* were obtained from CCLE database. **f** ESCC cells were treated with indicated concentrations of CYH33 for 1 h. Cell lysates were then subjected to Western blot for the indicated proteins

We have reported that the clinical PI3Kα-selective inhibitor CYH33 displayed potent activity against ESCC in vitro and in vivo.^18,19^ Though PI3K is frequently over-activated in ESCC,^20^ ESCC is highly heterogeneous in terms of molecular signatures. We screened the anti-proliferative activity of CYH33 and alpelisib, the marketed PI3Kα-selective inhibitor, in 24 lines of well characterized ESCC cells. As demonstrated in Fig. 1a, CYH33 significantly attenuated the proliferation of ESCC cells, yielding a median GI_50_ value of 0.47 μM. However, GI_50_ values varied dramatically from 0.08 μM (KYSE70) to 1.76 μM (KYSE140). Alpelisib displayed a similar pattern against these cell lines, with GI_50_ values 5 to 20-fold higher than those of CYH33. To identify the biomarker that potentially predicts the response to CYH33, the genomic alterations and gene expression at mRNA level were recruited from Cancer Cell Line Encyclopedia (CCLE) (https://depmap.org/portal/).^21^ Because *PIK3CA* mutation was utilized to stratify patients with hormone receptor-positive advanced breast cancer to receive alpelisib,^22^ the association between *PIK3CA* status and CYH33 activity was examined. Although KYSE510 and TE-5 cells harboring *PIK3CA*^*E545K*^ mutation were relatively sensitive to CYH33, there was no significant difference between cell lines carrying mutated or amplified *PIK3CA* and those without altered *PIK3CA* in terms of GI_50_ values (Fig. 1b). Similarly, the mRNA level of *PIK3CA* failed to show significant correlation with CYH33 activity in ESCC cells (Fig. 1c). Although amplification and overexpression of epidermal growth factor receptor (EGFR), an RTK sitting upstream of PI3K, was often identified in ESCC and associated with advanced stages and poor prognosis,^23^ alterations in EGFR failed to differentiate the sensitivity of ESCC cell lines to CYH33 (Fig. 1d). Likewise, no significant correlation was found between GI_50_ values and the mRNA level of *EGFR* in ESCC cells (Fig. 1e). Similarly, the status of *PIK3CA* or *EGFR* failed to predict alpelisib activity in ESCC cells (Supplementary Fig. S1a–d). We interrogated the effect of CYH33 on the PI3K signaling in sensitive (KYSE70 and KYSE510) and resistant (colo680N and KYSE30) cells. CYH33 suppressed the phosphorylation of Akt (S473) with a similar potency in both sensitive and resistant cells (Fig. 1f). CYH33 at the concentration of 12 nM or 37 nM potently decreased phosphorylated S6 in sensitive KYSE70 or KYSE510 cells respectively, while higher dose of CYH33 was needed to similarly inhibit the phosphorylation of S6K1 and S6 in resistant colo680N and KYSE30 cells (Fig. 1f). Thus, CYH33 exhibited potent while variable anti-proliferative activity against ESCC cells and *PIK3CA* status failed to predict its activity against ESCC cells.

### ESCC PDXs with *CCND1* amplification were more sensitive to CYH33

**Fig. 2:**
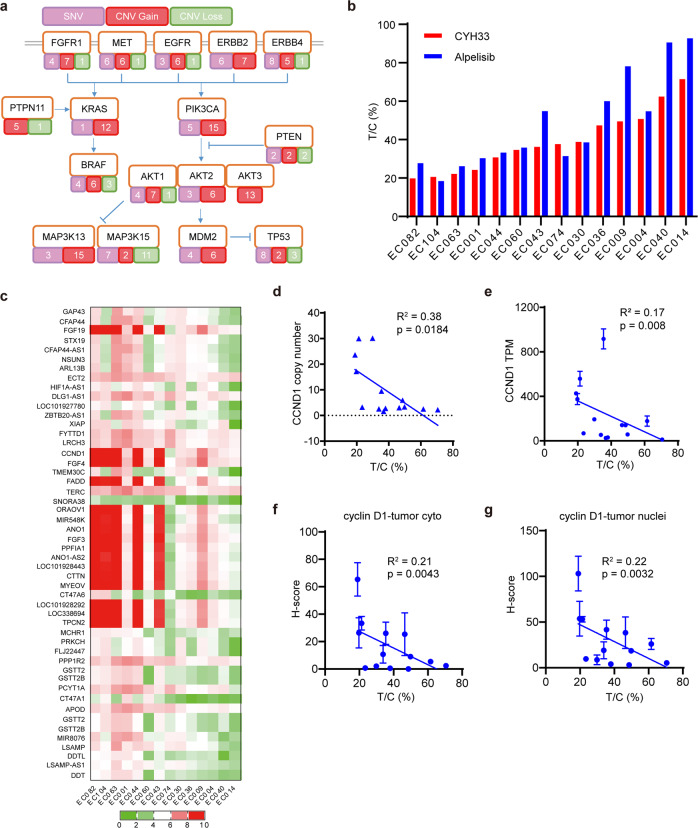
ESCC PDXs with *CCND1* amplification were more sensitive to CYH33. **a** Whole genome sequencing of 16 ESCC PDX samples. Alterations in RTK-PI3K pathway were presented indicating the number of PDX samples with SNV, CNV gain or CNV loss. **b** Randomly grouped BALB/c nude mice bearing ESCC PDXs were orally administered with vehicle (*n* = 12), CYH33 (25 mg/kg, once a day, *n* = 6) or alpelisib (50 mg/kg, once a day, *n* = 6). Tumor volume and body weight were measured twice per week, and the treatment to control ratios (T/C) were calculated at the end of treatment. **c** The correlation of the copy number of whole genome and T/C values obtained in 14 ESCC PDXs was analyzed. Top 50 genes with copy number negatively correlated with the T/C values were presented. **d**, **e** Pearson correlation analysis of the T/C values and *CCND1* copy number (**d**) or TPM values indicating *CCND1* mRNA level (**e**) of ESCC PDXs. **f**, **g** Expression level of cyclin D1 was measured by tissue microarray. Pearson correlation analysis of the T/C values and the H-score of cyclin D1 protein in tumor cytoplasm (**f**) or nucleus (**g**) was presented

To identify biomarkers indicative of CYH33 efficacy in more clinically relevant circumstances, we introduced a panel of 16 PDX models established from Chinese ESCC patients. We integrated single-nucleotide variations (SNVs) and copy number alterations from the whole-genome sequencing of the 16 ESCC cases. The median tumor mutational burden of the 16 PDXs is 3.2 mutations per megabase in protein-coding regions (Supplementary Fig. S2a), which is similar to that of 2.9 in 227 ESCC patients according to the TCGA database. Moreover, genomic alterations in important node genes involving in RTK and PI3K pathways were frequently detected (Fig. 2a). The genomic profile was consistent with those obtained from large-scale whole-genome sequencing or whole-exome sequencing of ESCC samples.^20^ Among these genes, *PIK3CA* was frequently altered, with 5/16 mutated and 15/16 amplified. Amplification of *KRAS* (12/16) and mutation or loss of *TP53* (8/16 and 3/16 respectively) were also identified (Fig. 2a). The efficacy of CYH33 (25 mg/kg/day) and alpelisib (50 mg/kg/day) were evaluated in 14 ESCC PDX models (EC008 and EC058 were not included in the evaluation due to their slow growth). CYH33 impeded the growth of most PDXs with treatment to control (T/C) values ranging from 18.98% in EC082 to 70.59% in EC014 (Fig. 2b & Supplementary Fig. S2b). Alpelisib displayed similar profile against the panel of ESCC PDXs. The T/C values were significantly correlated in two treatment groups (Supplementary Fig. S2c), which was consistent with that both compounds are PI3Kα inhibitors. PDXs with *PIK3CA*^*E545K*^ mutation (EC104, EC063, EC074, and EC030) were relatively sensitive to CYH33, with T/C values less than 40%. Meanwhile, several PDXs with wild-type *PIK3CA* (EC082, EC001, EC044, EC060, and EC043) were also sensitive to CYH33 treatment (T/C values less than 40%). The rest of PDXs harboring wild-type *PIK3CA* were relatively resistant to CYH33 (T/C values more than 45%) (Fig. 2b). These results indicated that although *PIK3CA* mutation might be able to distinguish part of sensitive ESCC patients, additional biomarkers are needed to predict the efficacy of PI3Kα inhibitors. We analyzed the correlation between T/C values and gene copy number of the whole genome. As shown in Fig. 2c, genes including *FGF19*, *CCND1*, *FGF4*, *FADD, etc*. located in chr11q13.3 region were highly amplified in CYH33-sensitive ESCC PDXs (Fig. 2c). As amplification of chr11q13.3 was frequently found in ESCC, we examined genes in this region more closely. *FGF3/4/19* encode fibroblast growth factor family members, which interact with fibroblast growth factor receptor and thus activate mitogen-activated protein kinase and PI3K signaling cascades.^24^ Although the copy number of *FGF3/4/19* negatively correlated with the T/C values (Supplementary Fig. S3a–c), *FGF3/4/19* were hardly expressed in ESCC samples according to data from GDC TCGA database accessed with UCSC Xena (http://xena.ucsc.edu/. Supplementary Fig. S3d).^25^ *CCND1* encodes cyclin D1, which sits downstream of PI3K pathway and was reported to be associated with the recurrence of ESCC.^26^ The copy number of *CCND1* was inversely related to the T/C values in PDX models (Fig. 2d). Elevated expression of cyclin D1 was also found in tumor tissues compared to normal counterparts in a cohort of ESCC patients (Supplementary Fig. S3d). Moreover, data from RNA sequencing and tissue microarray demonstrated that expression of *CCND1* at mRNA or protein level was negatively correlated with T/C values (Fig. 2e–g and Supplementary Fig. S3e). Thus, integrated multi-omic analysis indicated that ESCC PDXs with *CCND1* amplification tended to be sensitive to CYH33.

### G1 arrest was induced by CYH33 in sensitive cells via up-regulating p21

**Fig. 3:**
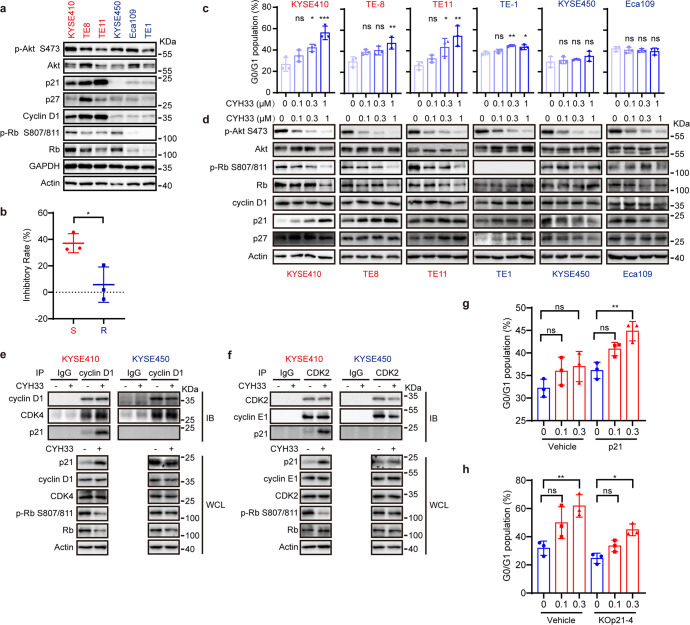
G1 arrest was induced by CYH33 in sensitive cells via up-regulating p21. **a** Cell lysates of sensitive and resistant ESCC cells were harvested and subjected to Western blot with the indicated antibodies. **b** Scatter plots of inhibitory rate of palbociclib (1 μM) grouped by sensitivity to CYH33 (*n* = 3). *p* value was determined by two-tailed unpaired Student’s *t*-test. *p < 0.05. **c**, **d** ESCC cells were treated with CYH33 at indicated concentrations for 24 h. Cell cycle distribution was analyzed with flow cytometry (**c**) and cell lysates were subjected to Western blot with indicated antibodies (**d**). **e**, **f** KYSE410 and KYSE450 cells were treated with CYH33 (1 μM) for 24 h, and immunoprecipitation with the antibody against cyclin D1 (**e**) or CDK2 (**f**) was performed followed by Western blot for the indicated antibodies. **g** KYSE450 cells transfected with plasmid expressing p21 or vehicle were treated with CYH33 for 24 h and cell cycle distribution was analyzed with flow cytometry. **h** *CDKN1A* was knocked out in KYSE410 cells by CRISPR. Monoclonal p21-KO cells (KOp21-4) and parental cells (Vehicle) were treated with CYH33 for 24 h and cell cycle distribution was analyzed with flow cytometry. Data were presented as mean ± SD (*n* = 3). *p* values were determined by two-tailed one-way ANOVA with Tukey multiple group comparison test. ns, *p* > 0.05; *p < 0.05; **p < 0.01

To explore the mechanism of cyclin D1 in determining the efficacy of the CYH33 in ESCC PDXs, we first analyzed the association between *CCND1* copy number and CYH33 activity in ESCC cell lines. Though most cell lines with *CCND1* copy number >2 were sensitive to CYH33 (except for TE9 and TE10 cell lines), there was no significant difference between cell lines with *CCND1* copy number >2 and those with *CCND1* copy number <2 in terms of GI_50_ values (Supplementary Fig. S4a). This appearing discrepancy might reflect the micro-environment in vitro and in vivo. It is well known that cyclin D1 binds to cyclin-dependent kinases (CDKs) including CDK4 and CDK6 and executes its oncogenic effect by promoting unlimited cell cycle progression.^27^ We thus selected three sensitive ESCC cell lines with *CCND1* copy number >2 and three resistant ESCC cell lines with *CCND1* copy number <2 to interrogate cell cycle pathway focusing on G1 phase regulation. As shown in Fig. 3a and Supplementary Fig. S4b, no significant difference in the level of phosphorylated Akt was found in sensitive cells (KYSE410, TE-8, and TE-11) and resistant cells (KYSE450, Eca109, and TE-1), while elevated expression of proteins in charge of G1/S transition, including cyclin D1, p21, and phosphorylated Rb was observed in sensitive cells compared to those in resistant cells, suggesting intact regulation at G1 phase in CYH33-sensitive cells. We evaluated the anti-proliferative activity of palbociclib, the first approved CDK4/6 inhibitor for the treatment of patients with HR-positve, HER2-negative breast cancer, in these 6 lines of ESCC cells. Palbociclib was significantly more potent against the proliferation of CYH33-sensitive cells with an average inhibitory rate of 37.1% than that of the resistant cells (inhibitory rate of 5.8% at the same concentration) (Fig. 3b). This observation indicated that CYH33-sensitive cells were more vulnerable to the blockade of G1/S transition. To determine whether CYH33 exerts its anti-proliferative activity by disturbing cell cycle progression, we examined cell cycle distribution after treatment of CYH33 for 24 h in this panel of ESCC cells. CYH33 significantly arrested cells at G0/G1 phase in sensitive cells in a concentration-dependent manner, while it possessed little effect in resistant cells (Fig. 3c). Accordingly, phosphorylation of Rb at S807/811 significantly decreased after CYH33 treatment in sensitive cells. No signal for phosphorylated Rb was detected in TE-1 cells because Rb is deficient in this cell line. Though cyclin D1 and the CDK inhibitor p27 remained largely unchanged in all tested cells, the CDK inhibitor p21 (alternatively p21^WAF1/Cip1^) accumulated in 3 lines of sensitive cells (Fig. 3d and Supplementary Fig. S4c). p21 slightly increased in TE-1 cells and even decreased in KYSE450 and Eca109 cells (Fig. 3d and Supplementary Fig. S4c). p21 acts as a brake in cell cycle progression by attenuating the activity of cyclin/CDK complexes to phosphorylate Rb. We measured the level of p21 in complex with cyclin D or cyclin E with immunoprecipitation. As shown in Fig. 3e and Supplementary Fig. S4d, p21 precipitated with cyclin D1 increased after exposure to CYH33 in sensitive KYSE410 cells but not in resistant KYSE450 cells. Similarly, p21 bound with CDK2-cyclin E1 complex elevated after CYH33 treatment in KYSE410 cells rather than in KYSE450 cells (Fig. 3f and Supplementary Fig. S4e). Thus, induction of p21 by CYH33 treatment resulted in enhanced binding of p21 with CDK4/6 and CDK2, which led to hypo-phosphorylation of Rb. To test whether overexpression of p21 could arrest resistant cells at G1 phase after exposure to CYH33, KYSE450 cells were transfected with plasmids expressing p21. As shown in Fig. 3g, ectopic expression of p21 enhanced G1 phase arrest induced by CYH33, which was accompanied with decreased phosphorylated Rb (Supplementary Fig. S4f) and elevated inhibition on cell proliferation (Supplementary Fig. S4g). By contrast, knockout *CDKN1A* that encodes p21 in sensitive KYSE410 cells restrained CYH33-induced G1 phase arrest as well as its anti-proliferative activity (Fig. 3h and Supplementary Fig. S4h, i). Taken together, CYH33 was more active in ESCC cells with intact regulation on G1/S transition, which was associated with induction of G1 phase arrest via accumulating p21 in sensitive ESCC cells.

### CYH33 accumulated p21 via blocking SKP2-mediated ubiquitination

**Fig. 4:**
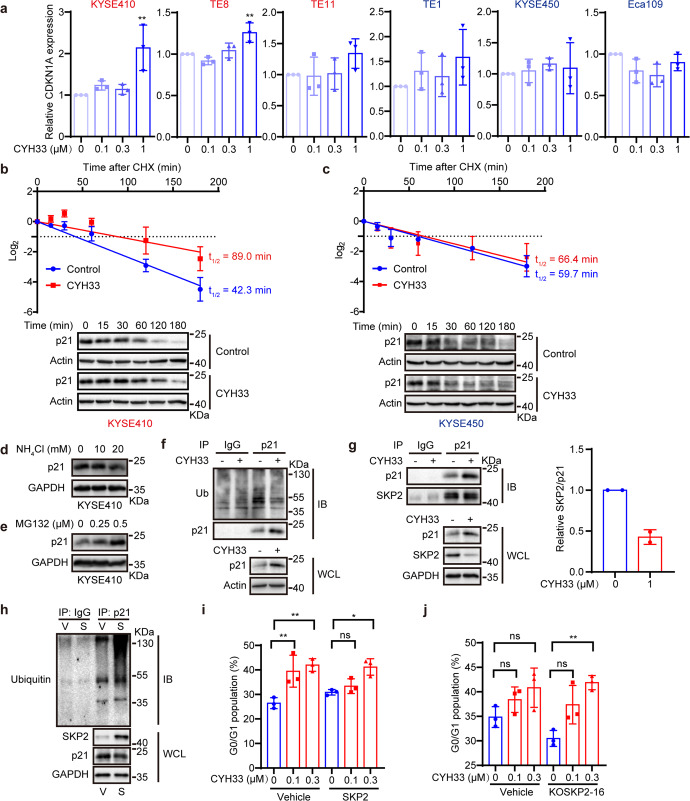
CYH33 accumulated p21 via blocking SKP2-mediated ubiquitination. **a** ESCC cells were treated with CYH33 at indicated concentrations for 24 h and mRNA level of *CDKN1A* was measured by quantitative real time PCR (*n* = 3). **b**, **c** KYSE410 (**b**, *n* = 3) and KYSE450 (**c**, *n* = 2) cells were pretreated with CYH33 (1 μM) or vehicle for 12 h, and then treated with CHX (25 μM) for indicated time. Cell lysates were subjected to Western blot with the indicated antibodies. The intensity of protein bands was quantified by Imagelab and the half-life of p21 was calculated after linear fitting. **d**, **e** KYSE410 cells were treated with NH_4_Cl (**d**) or MG132 (**e**) at indicated concentrations for 24 h. Cell lysates were subjected to Western blot with the indicated antibodies. **f** KYSE410 cells were treated with CYH33 (1 μM) for 24 h in the presence of MG132 (20 μM). Cell lysate was immunoprecipitated with the antibody against p21 and followed by Western blot with an anti-Ubiquitin antibody. **g** KYSE410 cells were treated with CYH33 (1 μM) for 24 h, and immunoprecipitation with the antibody against p21 was performed followed by Western blot for the indicated proteins. The bands were quantified by Imagelab (*n* = 2). **h** Ubiquitination of p21 was determined in KYSE450 cells transfected with plasmid expressing SKP2 (S) or vehicle (V) by immunoprecipitation with anti-p21 antibody followed by Western blot with anti-Ubiquitin antibody. **i** KYSE410 cells transfected with plasmid expressing SKP2 or vehicle were treated with CYH33 for 24 h and cell cycle distribution was analyzed with flow cytometry (*n* = 3). **j** *SKP2* gene was knocked out in KYSE450 cells by CRISPR. Monoclonal SKP2-KO cells (S2-16) and parental cells (Vehicle) were treated with CYH33 for 24 h and cell cycle distribution was analyzed with flow cytometry (*n* = 3). Data were presented as mean ± SD. *p* values were determined by two-tailed one-way ANOVA with Tukey multiple group comparison test. ns, *p* > 0.05; *p < 0.05; **p < 0.01

To investigate the mechanism of CYH33-induced p21 accumulation, we first determined the impact of CYH33 on *CDKN1A* mRNA level. CYH33 treatment for 24 h slightly elevated the mRNA level of *CDKN1A* at the concentration of 1 μM in part of sensitive ESCC lines (Fig. 4a), suggesting that there might be additional regulation of p21 at the protein level. We then measured the stability of p21 in KYSE410 and KYSE450 cells treated with CYH33 in the presence of cycloheximide (CHX). CYH33 prevented p21 from degradation with the half-life time (t_1/2_) prolonged from 42.3 min to 89.0 min in KYSE410 cells (Fig. 4b), while CYH33 hardly affected the t_1/2_ in KYSE450 cells (Fig. 4c). Moreover, inhibiting proteasome by MG132 but not inhibiting lysosome by NH_4_Cl significantly accumulated p21 in KYSE410 (Fig. 4d, e) and KYSE450 cells (Supplementary Fig. S5a, b). Accordingly, ubiquitination of p21 was suppressed after exposure to CYH33 in KYSE410 cells (Fig. 4f) but not in KYSE450 cells (Supplementary Fig. S5c). Several E3 ligases including S-phase kinase associated protein 2 (SKP2),^28^ STIP1 homology and U-box containing protein 1 (STUB1),^29^ cullin 4B (CLU4B)^30^ and makorin ring finger protein 1 (MKRN1)^31^ were reported to be involved in the ubiquitination and degradation of p21. The expression levels of these E3 ligases in normal esophagus tissues and ESCC tissues were examined. SKP2 stood out as the only one with significantly enhanced expression in ESCC tissues compared to normal counterparts (GSE23400, Gene Expression Omnibus, https://www.ncbi.nlm.nih.gov/geo/) (Supplementary Fig. S5d). Moreover, SKP2 has been found to participate in cell cycle regulation.^28,32,33^ We next detected the interaction of SKP2 and p21 in the presence of CYH33. As shown in Fig. 4g, CYH33 treatment resulted in accumulation of p21 which was accompanied with decreased SKP2. SKP2 was pulled down using an anti-p21 antibody, indicating the interaction of p21 and SKP2. However, CYH33 treatment impeded this interaction, illustrated by reduced ratio of SKP2 versus p21 pulled down by the anti-p21 antibody. To further confirm the role of SKP2 in regulating p21 stability, KYSE410 cells stably expressing SKP2 were established. Ectopic expression of SKP2 decreased the level of p21 accompanied with increased ubiquitination of p21 (Fig. 4h and Supplementary Fig. S5e). Moreover, forced expression of SKP2 abrogated CYH33-induced G1 phase arrest (Fig. 4i) and its anti-proliferative activity in KYSE410 cells (Supplementary Fig. S5f, g). By contrast, knockout *SKP2* in KYSE450 cells enhanced the anti-proliferative activity of CYH33 accompanied with enhanced arrest at G1 phase (Fig. 4j and Supplementary Fig. S5h, i). Thus, CYH33 induced accumulation of p21 and G1 phase arrest by alleviating SKP2-mediated ubiquitination of p21 in sensitive ESCC cells.

### CYH33 down-regulated the expression of SKP2 via attenuating E2F1-mediated transcription

**Fig. 5:**
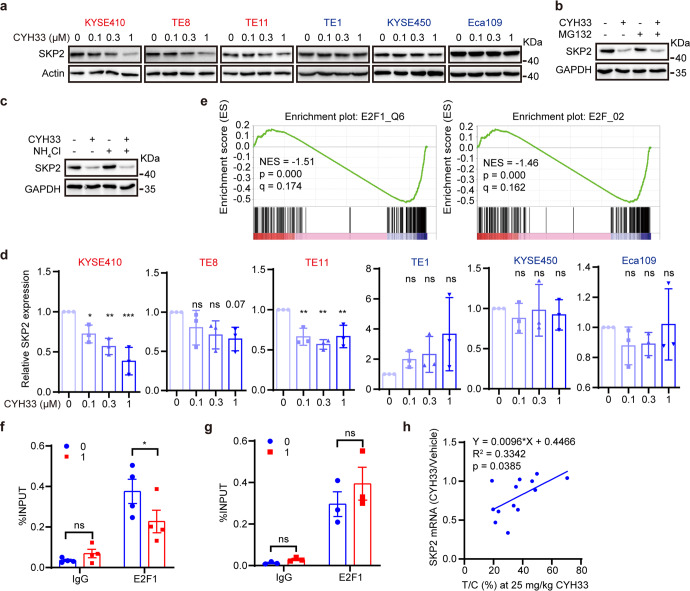
CYH33 down-regulated the expression of SKP2 via attenuating E2F1-mediated transcription. **a** ESCC cells were treated with CYH33 at indicated concentrations for 24 h and cell lysates were subjected to Western blot with the indicated antibodies. **b**, **c** KYSE410 cells were treated with CYH33 and MG132 (**b**) or NH_4_Cl (**c**) and cell lysates were subjected to Western blot with the indicated antibodies. **d** ESCC cells were treated with CYH33 at indicated concentrations for 24 h and the mRNA level of *SKP2* was measured by quantitative real time PCR (*n* = 3). **e** Gene set enrichment analysis was performed in KYSE410 cells treated with CYH33 (1 μM) for 24 h focusing on “C3 transcription factor targets”. Down-regulation of “E2F1_Q6” and “E2F_02” gene sets were presented. **f**, **g** ChIP assays were performed with the antibody against E2F1 in KYSE410 (**f**, *n* = 4) or KYSE450 (**g**, *n* = 3) cells treated with CYH33 (1 μM) for 24 h. Fold enrichment of E2F1 in the promoter region of *SKP2* was presented. **h** RNA sequencing was performed in ESCC PDX samples treated with CYH33. Pearson correlation analysis of the T/C values and the SKP2 mRNA level in CYH33 treatment group versus that in vehicle group was presented. Data were presented as mean ± SD. *p* values were analyzed using two-tailed one-way ANOVA with Tukey multiple group comparison test. ns, *p* > 0.05; *p < 0.05; **p < 0.01; ***p < 0.001

To determine whether the differential p21 degradation in sensitive and resistant cells was due to the distinct regulation of SKP2, we measured SKP2 protein after exposure to CYH33 for 24 h in the 6 lines of ESCC cells. CYH33 potently decreased the expression of SKP2 in a dose-dependent manner in sensitive KYSE410, TE-8 and TE-11 cells but not in resistant KYSE450, TE-1 and Eca109 cells (Fig. 5a). SKP2 only slightly decreased in KYSE450 cells upon exposure to 1 μM of CYH33 (Fig. 5a). However, neither inhibition of proteasome by MG132 nor inhibition of lysosome by NH_4_Cl rescued the reduction of SKP2 induced by CYH33 (Fig. 5b, c), indicating that decrease in SKP2 might not be due to loss of stability at protein level. The mRNA level of SKP2 was next examined in cells treated with CYH33. As shown in Fig. 5d, CYH33 reduced SKP2 mRNA dose-dependently in sensitive cells but not in resistant cells. The mRNA level of SKP2 even increased in CYH33-treated TE-1 cells. To identify transcriptional factors potentially regulate SKP2 transcription, we carried out Gene Set Enrichment Analysis focusing on transcription factor targets using gene expression profiles of KYSE410 cells treated with vehicle or CYH33 (1 μM) for 24 h. E2F family targeted genes were significantly down-regulated among the significantly enriched gene sets (Fig. 5e and Supplementary Fig. S6a, b). E2F1 has been reported to regulate the transcription of SKP2,^34^ which was verified by elevated activity of *SKP2* promoter after enforced expression of E2F1 in HEK293T cells (Supplementary Fig. S6c). We next conducted chromatin immunoprecipitation-quantitative polymerase chain reaction assay (ChIP-qPCR) using an antibody against E2F1. As shown in Fig. 5f and Supplementary Fig. S6d, CYH33 potently impeded the binding of E2F1 to the promoter region of SKP2 or CDC6, a known transcriptional target of E2F1, in sensitive KYSE410 cells. However, this observation failed to be obtained in resistant KYSE450 cells (Fig. 5g and Supplementary Fig. S6e). Moreover, the ratio of SKP2 mRNA level in CYH33 treatment group versus that in vehicle group was positively correlated with the T/C values in ESCC PDXs (Fig. 5h), indicating that ESCC PDXs with decreased SKP2 mRNA level upon CYH33 treatment were more sensitive to CYH33 treatment. Taken together, intact regulation of G1/S transition via the p21-Rb-E2F1-SKP2 loop mediated ESCC cells sensitive to PI3Kα inhibition.

### CDK4/6 inhibitors sensitized ESCC to CYH33

**Fig. 6:**
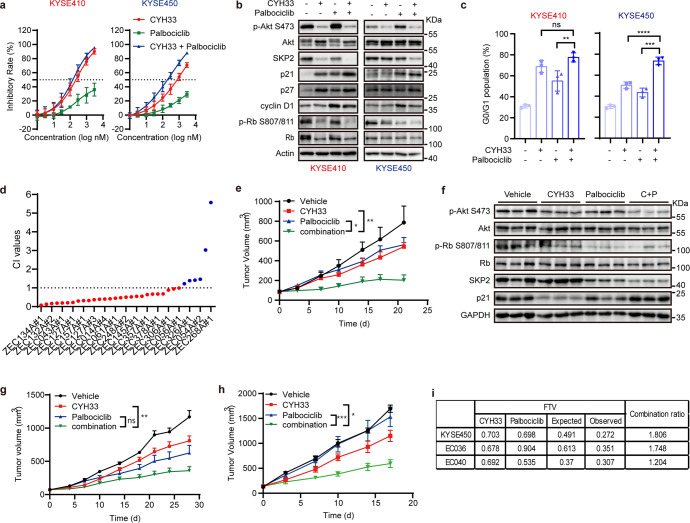
CDK4/6 inhibitors sensitized ESCC to CYH33. **a** KYSE410 and KYSE450 cells were treated with CYH33 and palbociclib alone or concurrently for 72 h. Cell proliferation was measured by SRB assay and combination index (CI) values were determined by CalcuSyn software (*n* = 3). **b**, **c** KYSE410 and KYSE450 cells were exposed to CYH33 (1 μM) and palbociclib (1 μM) alone or concurrently for 24 h. Cell lysates were then subjected to Western blot with the indicated antibodies (**b**) and cell cycle distribution was analyzed by flow cytometry (**c**, *n* = 3). Differences between the indicated groups were analyzed using two-tailed one-way ANOVA with Tukey multiple group comparison test. ns, *p* > 0.05; **p < 0.01; ***p < 0.001; ****p < 0.0001. **d** A panel of 33 lines of ESCC PDCs were treated with CYH33 and abemaciclib alone or concurrently in duplicate wells with a 9-concentration gradient for 72 h. Cell proliferation was assessed by CellTiter-Glo assay and CI values were determined by CalcuSyn software. **e**, **f** Randomly grouped BALB/c nude mice bearing KYSE450 xenografts were administrated orally with vehicle control, CYH33 (10 mg/kg), palbociclib (50 mg/kg), or a combination of CYH33 and palbociclib once a day for 21 days (*n* = 5). **e** Tumor volume was measured twice a week. Data were presented as mean + SEM. Differences between the indicated groups were analyzed using two-tailed one-way ANOVA with Tukey multiple group comparison test. *p < 0.05; **p < 0.01. **f** Three representative tumors from each group were homogenized and then subjected to Western blot with the indicated antibodies. **g**, **h** Randomly grouped BALB/c nude mice bearing ESCC PDX EC040 (**g**) or EC036 (**h**) were administrated orally with a vehicle control, CYH33 (10 mg/kg), palbociclib (50 mg/kg), or a combination of CYH33 and palbociclib once a day for 28 or 17 days (*n* = 6). Tumor volume was measured twice a week. Data were presented as mean + SEM. Differences between the indicated groups were analyzed using two-tailed one-way ANOVA with Tukey multiple group comparison test. ns, *p* > 0.05; *p < 0.05; **p < 0.01, ***p < 0.001. **i** The combination ratio of CYH33 and palbociclib in KYSE450, EC040, and EC036 xenografts was determined according to the Bliss independence model

Although CYH33 failed to arrest TE-1 cells at G1 phase due to truncated mutation (R787*) in *RB1* (Fig. 3c), CYH33 inhibited the proliferation of TE-1 cells, which were not responsive to CDK4/6 inhibitor palbociclib (Supplementary Fig. S7a). Thus, CYH33 may execute its anti-ESCC effect in both G1-phase arrest-dependent and -independent manners in sensitive cells. Failure to respond to CYH33-induced cell cycle arrest may render ESCC cells resistant to the treatment. To potentiate the activity of CYH33, sensitive KYSE410 cells and resistant KYSE450 cells were concurrently treated with the CDK4/6 inhibitor palbociclib and CYH33. As shown in Fig. 6a, palbociclib significantly sensitized resistant KYSE450 cells to CYH33 with a combination index (CI) value of 0.35, while the synergy was mild in sensitive KYSE410 cells with a CI value of 0.70 (Fig. 6a). To illuminate the mechanism of synergism between palbociclib and CYH33, we detected p21-Rb-E2F1-SKP2 loop and cell cycle distribution in KYSE410 and KYSE450 cells. CYH33 or palbociclib alone elevated p21 while decreased SKP2 accompanied by hypo-phosphorylation of Rb in KYSE410 cells. Combined treatment of CYH33 and palbociclib slightly enhanced these effects compared to single drug treatment (Fig. 6b). Consistently, the combined treatment failed to significantly enhance the cell population at G1 phase compared to CYH33 treatment alone (Fig. 6c). Meanwhile, palbociclib, but not CYH33, induced accumulation of p21 as well as decrease in SKP2 and phosphorylated Rb in CYH33-resistant KYSE450 cells. Concurrent treatment of CYH33 and palbociclib significantly potentiated these effects (Fig. 6b), and thus resulted in stronger G1-phase arrest in KYSE450 cells (Fig. 6c). Notably, combination of CYH33 with another marketed CDK4/6 inhibitor abemaciclib also displayed synergistic effect against proliferation in 27 out of 33 ESCC PDC cells (Fig. 6d). We then evaluated the efficacy of the combination in nude mice bearing KYSE450 xenografts. Palbociclib or CYH33 marginally inhibited the growth of KYSE450 xenografts with a T/C of 70.3% or 69.8% respectively, while concurrent administration of CYH33 and palbociclib synergistically attenuated tumor growth, yielding a T/C of 27.2% and a combination ratio of 1.806. (Fig. 6e, i and Supplementary Fig. S7b, c). Consistently CYH33 alone barely reduced the level of phosphorylated Rb or SKP2 in KYSE450 xenografts, while palbociclib suppressed phosphorylation of Rb and the level of SKP2. Concurrent administration of palbociclib and CYH33 potentiated the efficacy to inhibit Rb phosphorylation and SKP2 expression as well as to accumulate p21, indicating that the combination inhibited the cell cycle progression via p21-Rb-E2F1-SKP2 positive feedback loop in KYSE450 xenografts (Fig. 6f). We further tested the efficacy of the combination in CYH33-resistant ESCC PDXs EC036 and EC040, which harbored wild type *PIK3CA* and the copy number of *CCND1* were 3.0 and 2.5 respectively. Remarkably, palbociclib significantly sensitized EC036 and EC040 to CYH33 (Fig. 6g, h and Supplementary Fig. S7d–g), resulting in synergistic inhibition on the growth of PDXs (Fig. 6i). Thus, concurrent inhibition of CDK4/6 synergistically potentiated CYH33 against ESCC cells, PDCs and PDXs.

## Discussion

**Fig. 7:**
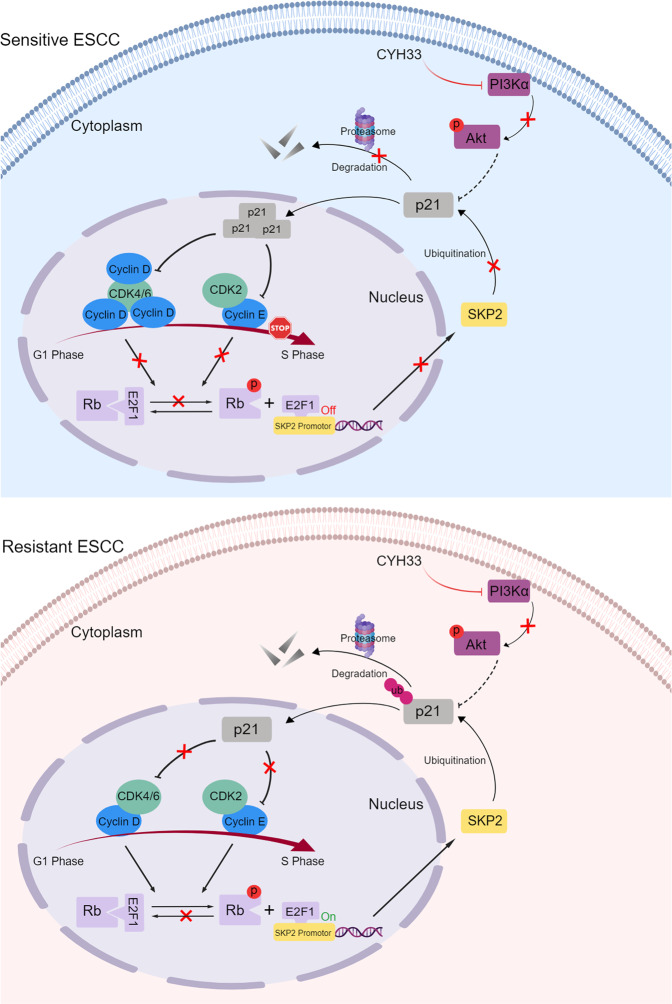
CYH33 arrested sensitive ESCC cells at G1 phase via the p21-Rb-E2F1-SKP2 positive feedback loop. A proposed scheme representing that the intact regulation on the G1/S transition via the p21-Rb-E2F1-SKP2 positive feedback loop determines the differential sensitivity to CYH33 in ESCC

Hyper-activation of PI3K pathway is frequently found in ESCC, making PI3K a potential target for treating ESCC. However, the response to PI3K inhibitors varies among ESCC patients, which spurs the identification of biomarkers that might predict or monitor the clinical efficacy. In this study, we revealed that ESCC PDXs with *CCND1* amplification were more sensitive to PI3Kα inhibitor CYH33. In addition, representative CYH33-sensitive ESCC cells displayed higher level of cyclin D1 as well as other regulators in charge of G1/S transition than resistant cells. CYH33 arrested sensitive ESCC cells in G1 phase by accumulating p21, which suppressed Rb phosphorylation by blocking the activity CDK4/6 and CDK2. Hypo-phosphorylation of Rb attenuated E2F1-mediated transcriptional activation of SKP2, which in turn hindered SKP2-mediated degradation of p21 and reinforced accumulation of p21. Our work suggests an important role of intact G1/S transition by p21-Rb-E2F1-SKP2 positive feedback loop in determining sensitivity to PI3Kα inhibitors in ESCC cells (Fig. 7). We also demonstrated that concurrent inhibition of CDK4/6 sensitized resistant ESCC cells and PDXs to CYH33.

*PIK3CA* mutation has been recommended as a sensitive biomarker for the approved PI3Kα inhibitor alpelisib in combination with fulvestrant for the treatment of HR-positive, HER2-negative advanced breast cancer.^13^ Accordingly, we found that ESCC PDXs with *PIK3CA* mutation were sensitive to CYH33. As the frequency of *PIK3CA* mutation is relatively low in ESCC, we identified *CCND1* amplification as a potentially additional biomarker indicating the efficacy of CYH33 for the therapy of ESCC. The copy number of *CCND1* was positively correlated with the efficacy of CYH33 in 14 tested ESCC PDXs. This correlation was also found in the expression of *CCND1* at mRNA and protein levels. *CCND1* locates in chr11q13.3 region, which is often amplified in ESCC. Moreover, the copy numbers of a cluster of genes located in the same region were also found to be positively correlated with the activity of CYH33. Thus, CYH33 displayed promising efficacy in ESCC with amplified chr11q13.3, which further provided rationale for the therapy of ESCC patients with PI3Kα inhibitors. Amplification of chr11q13.3 is also frequently found in breast cancer,^35^ head and neck squamous cell carcinoma,^36^ renal cell cancer,^37^ and lymph node metastasis.^38^ It is worthwhile to test PI3Kα inhibitors in these cancers with *CCND1* amplification. In consistency with the activity of cyclin D1 in G1 phase progression, we revealed that ESCC cells with intact regulation of G1/S transition were sensitive to CYH33. PI3K signaling pathway is important in regulating cell cycle transition from G1 to S phase^39,40^ and G1 phase arrest is one of the characteristics of inhibiting PI3K.^40^ However, inhibiting PI3K also attenuated the growth of tumor cells by reprogramming the metabolism. It has been reported that the metabolic features determined sensitivity to the PI3K/mTOR dual inhibitor gedatolisib in small cell lung cancer cells.^41^ We also found that CYH33 moderately inhibited the proliferation of ESCC cells without inducing cell cycle arrest, suggesting that CYH33 exerted its activity via both cell cycle-dependent and -independent manners. Meanwhile, the sensitivity of ESCC cells to CYH33 was associated with the induction of G1 phase arrest, suggesting that intact regulation of G1/S transition by PI3K might determine the sensitivity to CYH33. Cancer cells with elevated levels of cyclin D were highly sensitive to the CDK4/6 inhibition.^42^ We also found that the CDK4/6 inhibitor palbociclib displayed a similar profile while a less potency against ESCC cells compared to CYH33, further supporting the finding that intact G1/S transition rendered ESCC cells sensitive to CYH33. Our findings provided rationale to potentially extend the application of CYH33 for the treatment of ESCC with amplified *CCND1*. Nevertheless, these findings in ESCC cells and PDXS need to be verified in clinical settings, which might be fulfilled by the future studies with the data obtained from the ongoing clinical trials of CYH33 in ESCC patients (NCT03544905).

In an effort to dissect the mechanism of CYH33 to arrest ESCC cells at G1 phase, we revealed a p21-Rb-E2F1-SKP2 positive feedback loop rendering ESCC cells sensitive to CYH33. Blockade of PI3K signaling by CYH33 inhibited Rb phosphorylation in sensitive ESCC cells, which enabled the binding of Rb with E2F1 and suppressed E2F-mediated transcription of SKP2. Therefore, reinforced accumulation of p21 as a result of decreased ubiquitination by SKP2 further attenuated the phosphorylation of Rb by inhibiting both CDK4/6 and CDK2. Amplification of *CCND1* in ESCC cells suggests that the unlimited cell proliferation might be more dependent on CDK4/6, as these cells were more sensitive to CDK4/6 inhibition. However, CDK2 would circumvent the efficacy of CDK4/6 inhibitors and was reported to render resistance to CDK4/6 inhibitors in multiple cancer types.^43^ Initial inhibition of Rb phosphorylation would enhance the inhibition on CDK4/6 and CDK2 in tumor cells with intact regulatory loop by accumulating p21. Indeed, we found that breaking the loop by knockout p21 or overexpressing SKP2 attenuated the activity of CYH33 in sensitive cells, while enforcing the loop by overexpressing p21 sensitized resistant cells to CYH33. Moreover, decrease in the SKP2 mRNA level after CYH33 treatment was significantly correlated with its efficacy in ESCC PDXs. PI3K was reported to induce SKP2 expression via mTORC2^44^ and further investigations revealed that this regulation was mediated by E2F1-promoted SKP2 transcription in pancreatic ductal adenocarcinoma cells.^45^ Similarly, we found that inhibition of PI3K resulted in decreased binding of E2F1 to the promoter region of SKP2 and reduced expression of SKP2 in CYH33-sensitive ESCC cells. SKP2 has been recognized as an oncogenic protein, which promotes the cell cycle progression by facilitating the degradation of p21 and p27.^34^ Significant accumulation of p21 was observed in CYH33-sensitive ESCC cells, which indicated the crucial role of p21 in inducing G1 phase arrest upon PI3K inhibition. However, we could not rule out other mechanisms by which CYH33 inhibited tumor growth. For example, CYH33 treatment seemed to result in decreased cyclin D1 in sensitive PDXs (T/C < 40%) but not in resistant PDXs (T/C > 40%) (Supplementary Fig. S7h & i). This appearing discrepancy between sensitive ESCC cell lines and PDXs might due to the different treatment time and more complicated micro-environment in vivo. Moreover, it is unknown how the feedback loop was initially affected by PI3K inhibitors. Akt has been reported to regulate the stability of p21 by mediating phosphorylation at Thr145/Ser146 and relocation in cytoplasm.^46^ Though it appears that CYH33 possessed similar activity in inhibiting Akt phosphorylation in CYH33-sensitive and resistant ESCC cells, it’s worthwhile to further investigate the phosphorylation and location of p21 in CYH33-sensitive and -resistant cells.

PI3Kα inhibitors execute their anti-proliferative activity in both G1-phase arrest-dependent and -independent manners in sensitive cells. Therefore, concurrently inhibition of CDK4/6 could enhance the activity of CYH33 against resistant ESCC. Recently, three CDK4/6 inhibitors palbociclib, ribociclib, and abemaciclib were approved for the treatment of patients with HR-positive, HER2-negative breast cancer. We found palbociclib, the first approved CDK4/6 inhibitor, combined with CYH33 displayed synergistic effect in resistant KYSE450 cells and xenografts as well as two resistant ESCC PDX models. Combined CYH33 and abemaciclib also synergistically inhibited proliferation in 27 out of 33 ESCC PDC cells. These results indicated the important role of CDK4/6 in ESCC, which may also extend the application of CDK4/6 inhibitors. We have reported previously that simultaneous inhibition of PI3Kα and CDK4/6 synergistically suppressed KRAS-mutated non-small cell lung cancer.^47^ Concurrent targeting PI3K and cell cycle progression has displayed synergism in a variety of tumors, including breast cancer^48,49^ and lung squamous cell carcinoma,^50^ and attenuated ABCB1/P-gp mediated multi-drug resistance.^51^ We demonstrated that palbociclib extended the efficacy of CYH33 via suppressing phosphorylation of Rb and thus reinforcing the p21-Rb-E2F1-SKP2 loop. As monotherapy of PI3Kα displayed limited benefit in ESCC patients, it is worthwhile to evaluate the safety and efficacy of the combined regimen with CDK4/6 inhibitors for the treatment of ESCC with proficient Rb. Moreover, our findings also suggest SKP2 might be a potential therapeutic target for ESCC by stabilizing p21 and blocking cell cycle progression.

In summary, we found that *CCND1* amplification was a potential biomarker, which might indicate intact regulation of G1/S transition and predict sensitivity to PI3Kα inhibitors in ESCC. CYH33 attenuated cell cycle progression via the p21-Rb-E2F1-SKP2 feedback loop, and co-targeting CDK4/6 enhanced CYH33 activity against ESCC cells and PDXs. These findings provided mechanistic rationale to test PI3Kα inhibitor in ESCC patients with amplified *CCND1* and its combination with CDK4/6 inhibitors for the therapy of ESCC with proficient Rb.

## Materials and methods

### Study approval

All animal experiments were carried out according to the Institutional Ethical Guidelines on Animal Care and were approved by the Institute of Animal Care and Use Committee at Shanghai Institute of Materia Medica.

### Cell lines and cell culture

The ESCC cells KYSE70, KYSE150, KYSE180, KYSE270, KYSE410, KYSE510, KYSE450, KYSE30, and KYSE140 were kindly provided by Dr. Hideaki Shimada (Department of Surgery, Toho University School of Medicine). The ESCC cells TE-14, TE-6, TE-8, OE-21, TE-5, TE-11, T.T, TE-15, TE-4, EC-GI-10, TE-1, TE-10, and TE-9 were from RIKEN Cell Bank (established by Dr. Nishihira, Tetsuro). Cell lines employed were authenticated by analyzing short tandem repeats at Genesky Biotechnologies Inc. (Shanghai, China). Cells were cultured with recommended medium in a humidified atmosphere containing 5% CO_2_ at 37 °C.

### Compounds

CYH33 was obtained from Shanghai HaiHe Biopharma Co., Ltd (Shanghai, China). Alpelisib was purchased from Selleck Chemicals (Houston, USA). Palbociclib was from Meilunbio (Dalian, China). For experiments in vitro, all compounds were dissolved in dimethyl sulfoxide (DMSO, Sigma, St. Louis, USA) at 10 mM and stored at −20 °C. For animal studies, palbociclib was dissolved in normal saline, while alpelisib and CYH33 were dissolved in normal saline containing 0.5% of Tween 80 (v/v; Sangon Biotech, Shanghai, China) and 1% of CMC-Na (m/v; Sinopharm, Beijing, China).

### Cell proliferation assay

Sulforhodamine B (SRB) assay was employed to evaluate cell proliferation as described previously.^52^ The proliferation of patient-derived cells (PDCs) of ESCC was conducted by 3D Medicines (Shanghai, China) measured with CellTiter-Glo assay (Promega Corporation, Madison, USA). The inhibitory rate was calculated using the formula: (OD_control cells_ − OD_treated cells_) / OD_control_ _cells_ × 100% or (OD_control_ _cells_ − OD_treated cells_) / (OD_control cells_ − OD_Day0 cells_) × 100%. IC_50_ or GI_50_ values were computed by four parameter concentration response curves fitting with SoftMaxPro (Molecular Devices, USA).

### Flow cytometry

Cell cycle distribution was analyzed as described previously.^52,53^ Data were obtained with an ACEA NovoCyte^TM^ (ACEA Biosciences, San Diego, USA) and analyzed with NovoExpress software.

### Western blotting

Cells or tumor tissues were lysed with radioimmunoprecipitation assay buffer (Beyotime, Shanghai, China, #P0013B) supplemented with protease inhibitors and phosphatase inhibitors (Beyotime, Shanghai, China, #P1006). Standard Western blotting was performed with antibodies against Akt (#4691), phospho-Akt (Ser473; #4060), S6 (#2217), phospho-S6 (Ser235/236; #4858), phospho-S6 (Ser240/244; #5364), S6K (#5707), phospho-S6K (Thr389; #9205), cyclin D1 (#55506), p21 (#2947), p27 (#3686), Rb (#9313), phosphor-Rb (Ser807/811; #8516), CDK6 (#13331), CDK4 (#12790), CDK2 (#18048), cyclin E1 (#20808), SKP2 (#2652) (Cell Signaling Technology, Danvers, USA) and β-Actin (#A5441), GAPDH (#G8795) (Sigma, St. Louis, USA). Images were captured with the ChemiDoc Touch Imaging System (Bio-Rad, California, USA).

### Next-generation sequencing

Genomic DNA and total RNA was isolated and purified using AllPrep DNA/RNA mini Kit (Qiagen, Dusseldorf, Germany). For the sequencing of genomic DNA, paired-end libraries were constructed with insert size of approximately 400 bp using TruSeq Nano DNA Library Prep Kit (Illumina, USA). The constructed libraries were sequenced with the Illumina HiSeq X Ten sequencing system. Sequence reads were mapped onto hg19 reference genome with BWA (v0.7.9a). The alignment results were used to call single nucleotide variants (SNVs) with GATK pipeline (v3.4). CNVkit (v0.8.3) was employed to determine copy number variations. RNA-Seq libraries were constructed using pair-end adapters with an Illumina mRNA sequencing kit. The libraries were sequenced with the Illumina HiSeq X Ten sequencing system. Transcript quantification and differential expression analysis were performed using Salmon (v0.8.0) and DESeq2, respectively.

### Colony-formation assay

ESCC cells in single-cell suspension were planted in 6-well plates and incubated for about 10 days to form colonies. Colonies were fixed with methanol and stained with crystal violet (0.1% w/v). Colonies were photographed with ChemiDoc Touch Imaging System (Bio-Rad, Hercules, USA).

### Quantitative RT-PCR

RNA was extracted with TRIzol (Thermo Fisher Scientific) and was reverse-transcribed with HiScript II Q Select RT SuperMix (Vazyme, #R233-01). PCR was conducted utilizing iQ SYBR Green Supermix (Bio-Rad, Hercules, USA) with the CFX realtime PCR system (Bio-Rad). *ACTB* was used as internal control. Primers employed were as follows: ACTB-F (5′-CATGTACGTTGCTATCCAGGC-3′), ACTB-R (5′-CTCCTTAATGTCACGCACGAT-3′), SKP2-F (5′- ATGCCCCAATCTTGTCCATCT-3′), SKP2-R (5′- CACCGACTGAGTGATAGGTGT-3′), CDKN1A-F (5′- TGTCCGTCAGAACCCATGC-3′), and CDKN1A-R (5′- AAAGTCGAAGTTCCATCGCTC-3′).

### Chromatin immunoprecipitation (ChIP)

ChIP assays were conducted using the SimpleChip Plus Enzymatic Chromatin IP Kit (Cell Signaling Technology, Danvers, USA, #9005) with the antibody against E2F1 (Cell Signaling Technology, #3742) as instructed by the manufacturer. Immunoprecipitated DNA were determined by PCR using indicated primers: SKP2-F (5′-CTCCCCGCCTACCCCGTGG-3′), SKP2-R (5′-CAGACCCGCTAAGCCTAGCAACG-3′), CDC6-F (5′-AAAGGCTCTGTGACTACAGCCAAT-3′) and CDC6-R (5′-GTGCAGGATCCTTCTCACGTCTCTCAC-3′).

### Immunoprecipitation

Cells were lysed with the buffer supplemented with 1% NP-40 (Beyotime, Shanghai, China, #P0013F). 1 mL of cell lysate (1 mg protein/mL) was incubated with 5 μg of indicated antibody or normal rabbit IgG overnight at 4 °C. On the next day, 10 μl protein A/G agarose was added and further incubated for 2 h. The protein A/G agarose was pelleted with a magnet and washed 3 times. Immuno-complexes were eluted with loading buffer by boiling for 10 min and subjected to Western blotting.

### Plasmid construction

Human *CDKN1A* or *SKP2* was cloned and ligated into the expressing vector pCDH-CMV-MCS-EF2 purchased from Synbio Technologies (Suzhou, China). Single guide RNA targeting *CDKN1A* or *SKP2* was annealed and cloned into LentiCRISPR v2 plasmid (Addgene, #52961). The sequences for sgRNA were as follows: SKP2-1 (5′- CACCGGCAACGTTGCTACTCAGGTC-3′), SKP2-2 (5′- AAACGACCTGAGTAGCAACGTTGCC-3′), CDKN1A-1 (5′- CACCGAGTCGAAGTTCCATCGCTCA-3′), and CDKN1A-2 (5′- AAACTGAGCGATGGAACTTCGACTC-3′).

### Virus production and cell infection

HEK293T cells were transfected with plasmids of interest, psPAX (Addgene, #12260) and pMD2.G (Addgene, #12259) using Lipofectamine 2000 (Invitrogen, Carlsbad, USA) as the protocol provided by the manufacturer. Medium supernatant containing lentivirus was collected with a 0.45 μm filter 48 h after transfection. ESCC cells were infected with the virus with the help of polybrene at 6 μg/mL (Sigma, St. Louis, USA). Cells were selected in the presence of of puromycin (3 μg/mL).

### Animal studies

BALB/c athymic nude mice aged 4–5-weeks were obtained from the Shanghai Institute of Materia Medica (Shanghai, China). ESCC PDXs were kindly provided by Zhongshan hospital (Shanghai, China). Xenografts derived from KYSE450 cells were established by subcutaneously injecting cells suspended in Matrigel into the right side of axillary. Tumor sections were cut into pieces of about 40 mm^3^ and then transplanted subcutaneously into mice. When tumor volume reached about 150 mm^3^, mice were randomized and administered orally with vehicle control or indicated compounds once a day. Body weight was recorded with an electronic balance and tumor volume was measured using a microcaliper twice per week. The tumor volume (V) was calculated using the formula V = a^2^b/2, and a and b represented the tumor’s width and length respectively. RTV was calculated by the formula RTV = V_t_/V_0_, where V_0_ represented the tumor volume at the beginning of treatment, and V_t_ represented the tumor volume upon treatment. Treatment to control (T/C) values were calculated using the formula T/C = RTV_treatment_/RTV_control_ × 100%. All mice were housed in a specific pathogen-free facility at Shanghai Institute of Materia Medica Animal Resource Center. Mice were maintained under a 12-h light–dark cycle with free access to food and water.

### Combination analysis

The combinatorial effect in vitro was analyzed by CalcuSyn software (Biosoft, Cambridge, UK) to determine the combination index (CI).^54^ A CI = 1 indicated an additive effect, a CI > 1 indicated antagonism, and a CI < 1 indicated synergism. The combinatorial effect in vivo was evaluated by combination ratio with the Bliss independence model.^55,56^ Synergy, additive effect or antagonism was defined when the combination ratio was more than 1, 1, or lower than 1 respectively.

### Tissue microarray and immunohistochemical staining

The esophageal carcinoma tissue microarray was constructed by extracting 2 mm diameter cores of PDX tumor paraffin blocks and re-embedding into a gridded paraffin block. The immuno-staining was performed using antibody against cyclin D1 (Cell Signaling Technology, #2978) as previously described.^57^ Stained slides were imaged using the Vectra 2.0 quantitative pathology imaging system (PerkinElmer, Waltham, USA). Acquired image files were spectrally unmixed and subjected to tumor/stromal tissue segmentation, nulcei/cytoplasm segmentation and 4-bin (0–3+) scoring using inform software (PerkinElmer, Waltham, USA). H-score was calculated by adding the percentage of strongly stained (×3), moderately stained (×2), and weakly stained cells (×1) as described.^58^

### Statistics

Experiments were repeated at least three times or otherwise stated, and data were presented as mean ± standard deviation (SD) or mean ± standard error of mean (SEM). Data were tested for normality using the Shapiro-Wilk test. Statistical analyses were performed using Prism 8 (GraphPad, La Jolla, USA). Statistical comparison was carried out with Student’s *t* test for two groups or one-way ANOVA followed by Tukey multiple group comparison tests for more than two groups. *p < 0.05, **p < 0.01, ***p < 0.001.

## Supplementary information

supplementary materials-clean

## Data Availability

All research data supporting the findings of this study are available upon reasonable request by readers. The raw data of genomic-seq and RNA-seq have been deposited in the SRA (PRJNA909939) and GEO database (GSE162658) respectively.
