# Supplementary material for: Intact regulation of G1/S transition renders esophageal squamous cell carcinoma sensitive to PI3Kα inhibitors
Source: Signal Transduct Target Ther. 2023 Apr 12;8:153. doi: 10.1038/s41392-023-01359-x (PMC10090078; doi:10.1038/s41392-023-01359-x)
Supplement: Supplementary file 1 — supplementary materials-clean [file 41392_2023_1359_MOESM1_ESM.docx]

Supplementary Materials for

Intact regulation of G1/S transition renders esophageal squamous cell carcinoma sensitive to PI3Kα inhibitors

Xu Zhang^1,2#^, Yuxiang Wang^1#^, Xi Zhang^1^, Yanyan Shen^1^, Kang Yang^3^, Qingyang Ma^4^, Yuemei Qiao^5^, Jiajie Shi^1^, Yi Wang^1^, Lan Xu^1^, Biyu Yang^1^, Gaoxiang Ge^5^, Landian Hu^4^, Xiangyin Kong^4^, Chunhao Yang^6^, Yi Chen^1,2*^, Jian Ding^1,2*^, Linghua Meng^1,2,3*^

Correspondence to: Yi Chen (ychen@simm.ac.cn), Jian Ding (jding@simm.ac.cn), Linghua Meng (lhmeng@simm.ac.cn)

**This PDF file includes:**

Materials and Methods

Figures. S1 to S7

Materials and Methods

Luciferase reporter assay

The promoter region of Human SKP2 were cloned by PCR and ligated into the pGL4.10 vector (Promega, Madison, USA). cDNA of Human E2F1 was cloned by RT-PCR and ligated into the expressing vector pcDNA3.1-EGFP (Synbio Technologies, Suzhou, China). HEK293T cells was transfected with SKP2-pGL4.10, pGL4.74 (Promega, Madison, USA), and control vector or E2F1 expressing plasmid. pGL4.74 expressing Renilla luciferase was used as a control. Relative luciferase activity was analyzed using a Dual-Glo Luciferase Reporter Assay Kit (Meilunbio, Dalian, China) at 48 h post transfection. The primers used for cloning were as follows: SKP2-promotor-F (5’- GGGGTACCCTTAGAATTCATCCCCCTTCCCT -3’), SKP2-promotor-R (5’- CCGCTCGAGATTTCATGCTCTTCCCCTTTTT -3’); E2F1-F (5’- CTAGCTAGCATGGCCTTGGCCGGGGCCCCT -3’), and E2F1-R (5’- CGGGATCCTCAGAAATCCAGGGGGGTGAGG -3’).


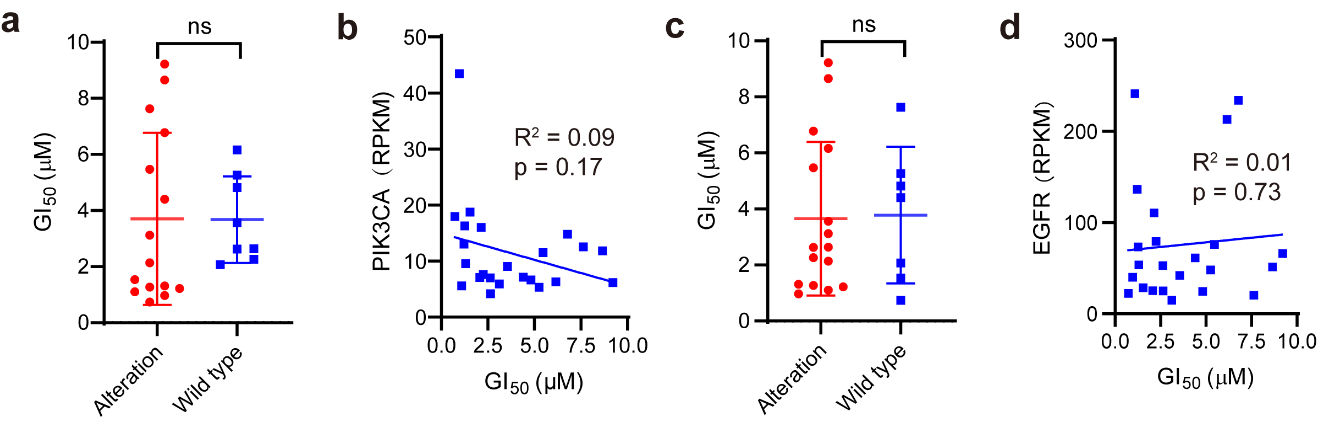


Figure. S1. Alpelisib displayed potent while variable anti-proliferative activity in ESCC cells.

(a, c) Scatter plots of mean GI_50_ values of alpelisib in cells grouped by alterations in *PIK3CA* (a) and *EGFR* (c). Difference between groups was analyzed by two-tailed unpaired Student’s *t*-test. ns, p > 0.05. (b, d) Pearson correlation analysis of the mean GI_50_ values of alpelisib and RPKM values of *PIK3CA* (b) and *EGFR* (d) was presented. RPKM values of *PIK3CA* and *EGFR* were obtained from CCLE database.


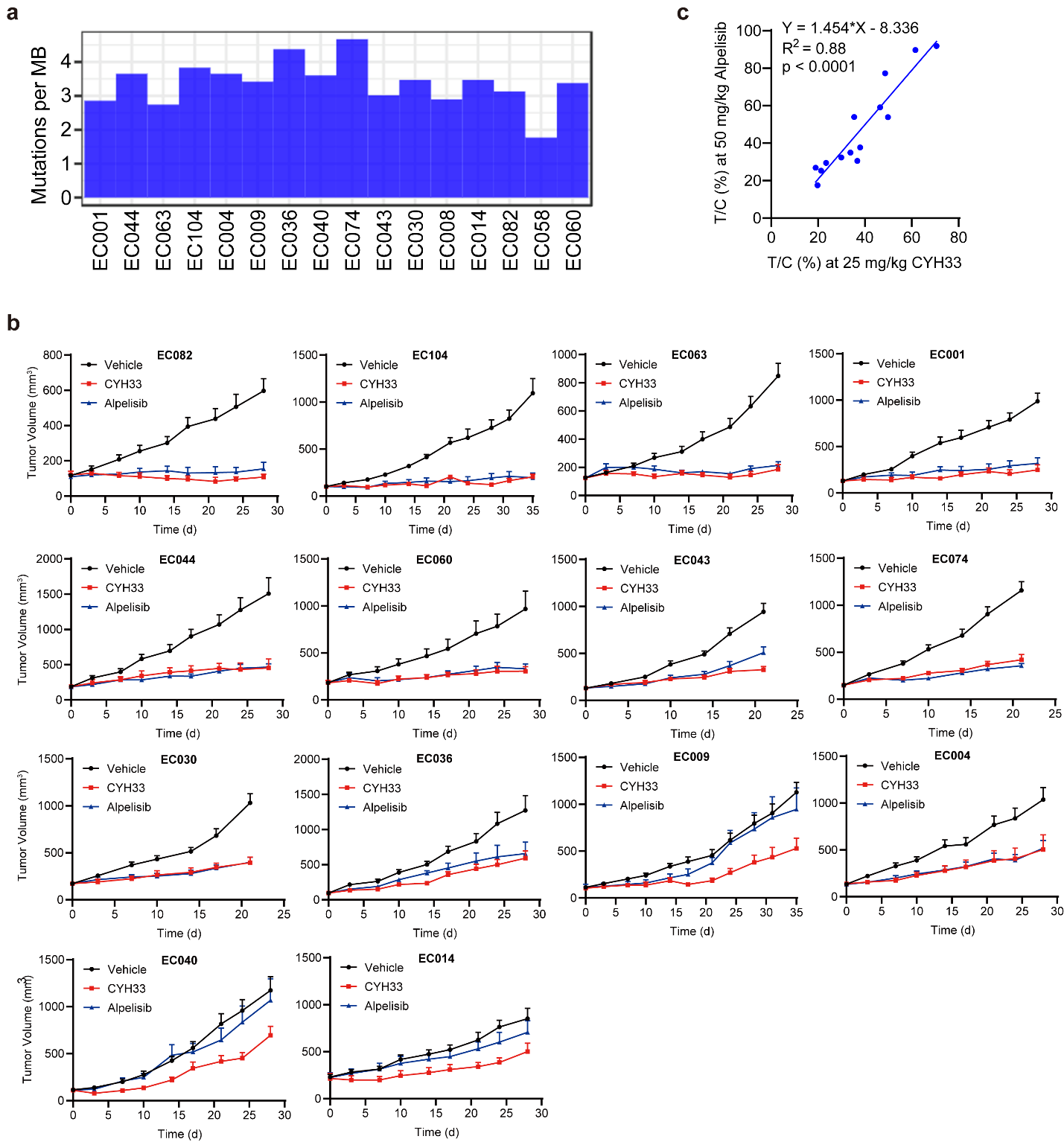


Figure. S2. PI3Kα inhibitors displayed potent while variable activity against the growth of ESCC PDXs.

(a) Tumor mutational burden of the 16 ESCC PDXs were displayed as mutations per megabase. (b) Randomly grouped BALB/c nude mice bearing ESCC PDXs were orally administered with CYH33 (25 mg/kg) or alpelisib (50 mg/kg) once a day. Tumor volume and body weight were measured twice per week. (c) Pearson correlation analysis of the T/C values obtained after treatment of CYH33 (25 mg/kg) or alpelisib (50 mg/kg).


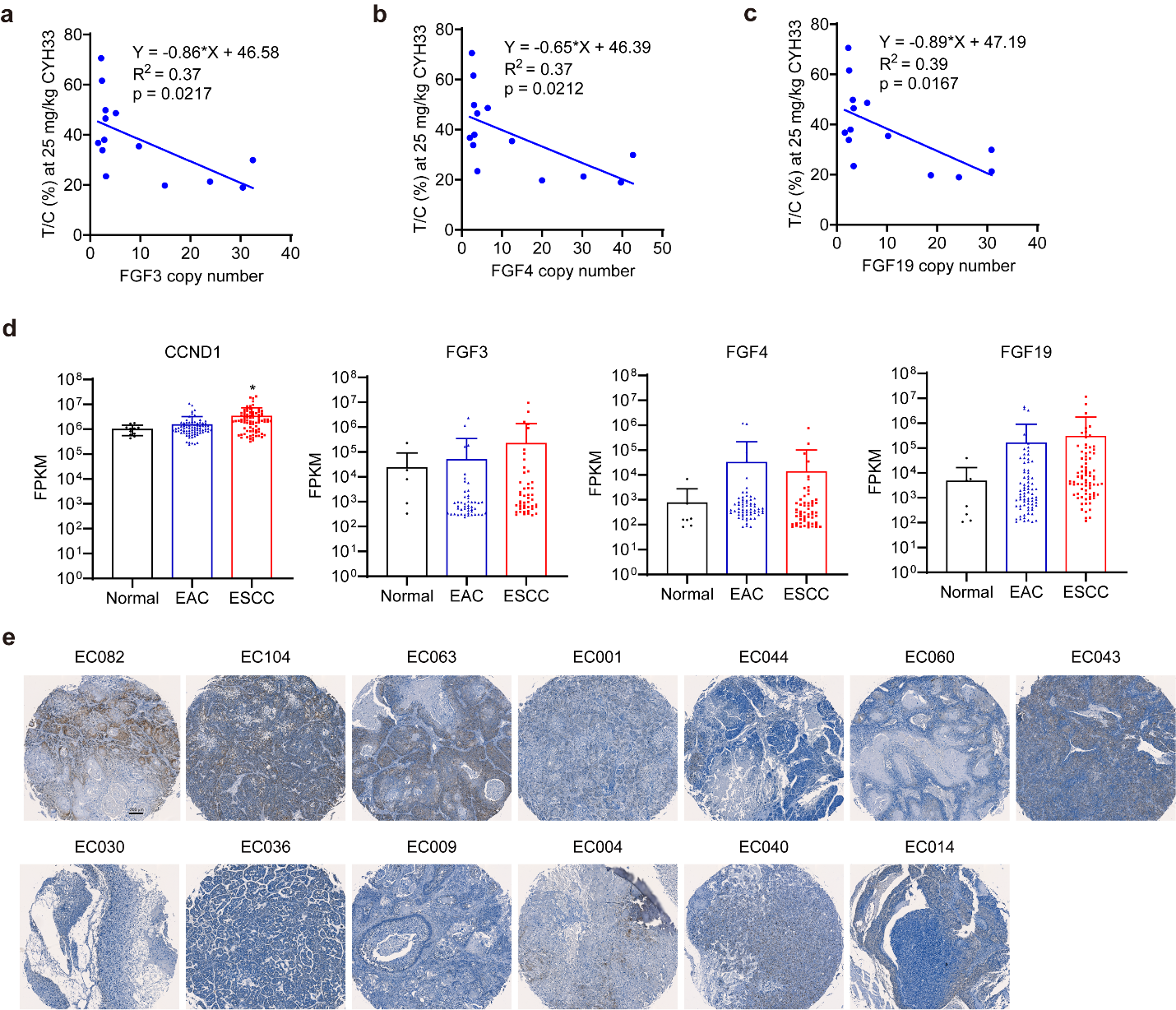


Figure. S3. ESCC PDXs with *CCND1* amplification were more sensitive to CYH33.

(a-c) Pearson correlation analysis of the T/C values and copy numbers of *FGF3* (a), *FGF4* (b), and *FGF19* (c) in ESCC PDXs. (d) TCGA data were analyzed using UCSC Xena. Presented was expression level of *CCND1*, *FGF3*, *FGF4*, and *FGF19* mRNA in esophageal tissues, esophageal adenocarcinoma, and ESCC. Differences between the indicated groups were analyzed using two-tailed one-way ANOVA with Tukey multiple group comparison test. *, p < 0.05. (e) Representative images of immunohistochemistry staining of cyclin D1 in tumor tissues of 13 ESCC PDXs derived from Chinese ESCC patients. Scale bar: 200 μm.


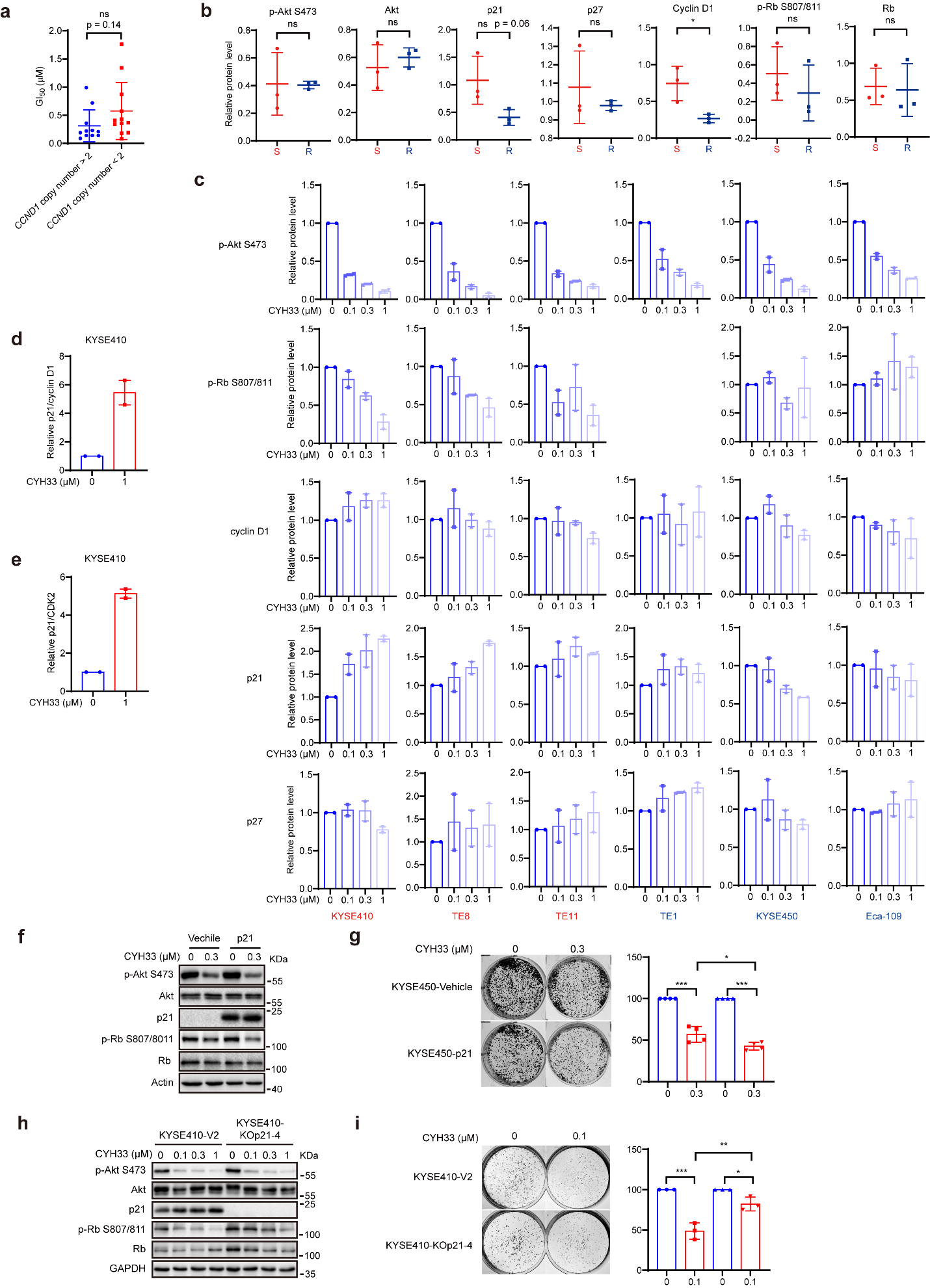


Figure. S4. CYH33 induced G1 arrest in sensitive cells via up-regulating p21.

(a) Scatter plots of mean GI_50_ values of CYH33 in cells grouped by *CCND1* copy number. Difference between groups was analyzed by two-tailed unpaired Student’s t-test. ns, p > 0.05. The data of *CCND1* copy number were obtained from CCLE database. (b) Scatter plots of relative protein level in Figure 3a grouped by sensitivity to CYH33 (n = 3). p value was determined by two-tailed unpaired Student’s *t*-test. ns, p > 0.05; *, p < 0.05. (c-e) The bands in Figure 3d (c), Figure 3e (d) and Figure 3f (e) were quantified by Image lab (n = 2). (f-g) KYSE450 cells were transfected with plasmid expressing p21 or vehicle. (f) Cells were treated with CYH33 (0.3 μM) for 24 h and cell lysates were subjected to Western blot with the indicated antibodies. (g) Colony formation assay in the presence of CYH33 (0.3 μM) for 10 d (n = 4). The colonies were quantified by Image J software. Data were presented as mean ± SD. Differences between the indicated groups were analyzed using two-tailed one-way ANOVA with Tukey multiple group comparison test. *, p < 0.05; ***, p < 0.001. (h-i) *CDKN1A* was knocked out in KYSE410 cells by CRISPR. (h) Monoclonal p21-KO cells (KOp21-4) and parental cells (V2) were treated with CYH33 for 24 h and cell lysates were subjected to Western blot with the indicated antibodies. (i) Colony formation assay in the presence of CYH33 (0.1 μM) for 10 d (n = 3). The colonies were quantified by Image J software. Data were presented as mean ± SD. Differences between the indicated groups were analyzed using two-tailed one-way ANOVA with Tukey multiple group comparison test. *, p < 0.05; **, p < 0.01; ***, p < 0.001.


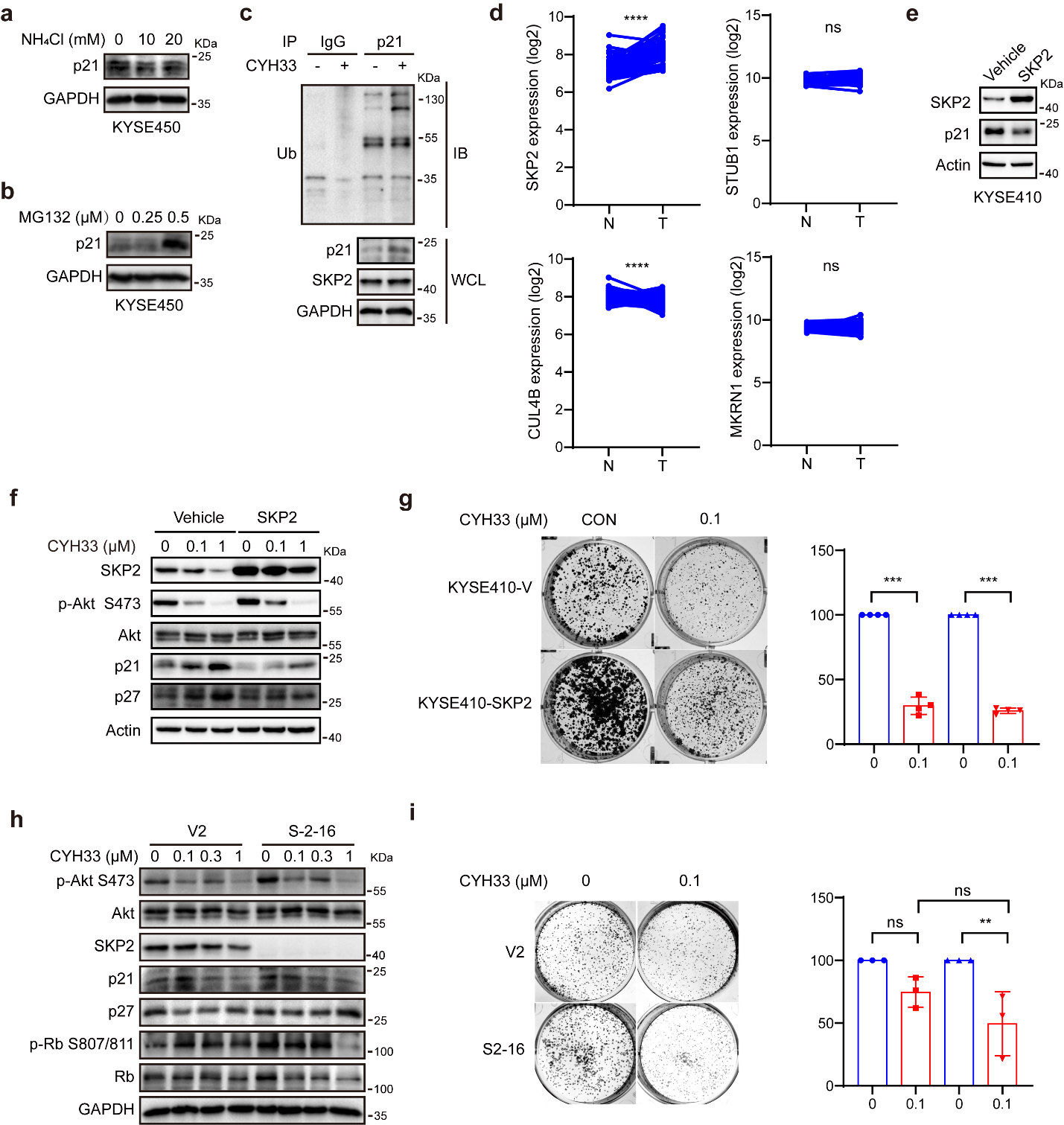


Figure. S5. CYH33 accumulated p21 via blocking SKP2-mediated ubiquitination.

(a-b) KYSE450 cells were treated with NH_4_Cl (a) or MG132 (b) at indicated concentrations for 24 h. Cell lysates were subjected to Western blot with the indicated antibodies. (c) KYSE450 cells were treated with CYH33 (1 μM) for 24 h in the presence of MG132 (20 μM). Cell lysate was immunoprecipitated with the antibody against p21 and followed by Western blot with an anti-Ubiquitin antibody. (d) mRNA data of SKP2, STUB1, CUL4B and MKRN1 in ESCC tissues (n = 53) and adjacent normal tissues (n = 53) were retrieved from the Gene Expression Omnibus database (https://www.ncbi.nlm.nih.gov/geo/, GSE23400). Difference between groups was analyzed by Wilcoxon matched-pairs signed rank test. ns, p > 0.05; ****, p < 0.0001. (e) KYSE410 cells transfected with plasmid expressing SKP2 or vehicle were lysed, and cell lysates were subjected to Western blot with the indicated antibodies. (f-g) KYSE410 cells were transfected with plasmids expressing SKP2 or vehicle. (f) Cells were treated with CYH33 for 24 h and cell lysates were subjected to Western blot with the indicated antibodies. (g) Colony formation assay in the presence of CYH33 (0.1 μM) for 10 d (n = 4). The colonies were quantified by Image J software. Data were presented as mean ± SD. Differences between the indicated groups were analyzed using two-tailed one-way ANOVA with Tukey multiple group comparison test. ***, p < 0.001. (h-i) *SKP2* gene was knocked out in KYSE450 cells by CRISPR. (h) Monoclonal SKP2-KO cells (S2-16) and parental cells (V2) were treated with CYH33 at indicated concentrations for 24 h and cell lysates were subjected to Western blot with the indicated antibodies. (i) Colony formation assay in the presence of CYH33 (0.1 μM) for 10 d (n = 3). The colonies were quantified by Image J software. Data were presented as mean ± SD. Differences between the indicated groups were analyzed using two-tailed one-way ANOVA with Tukey multiple group comparison test. ns, p > 0.05; **, p < 0.01.


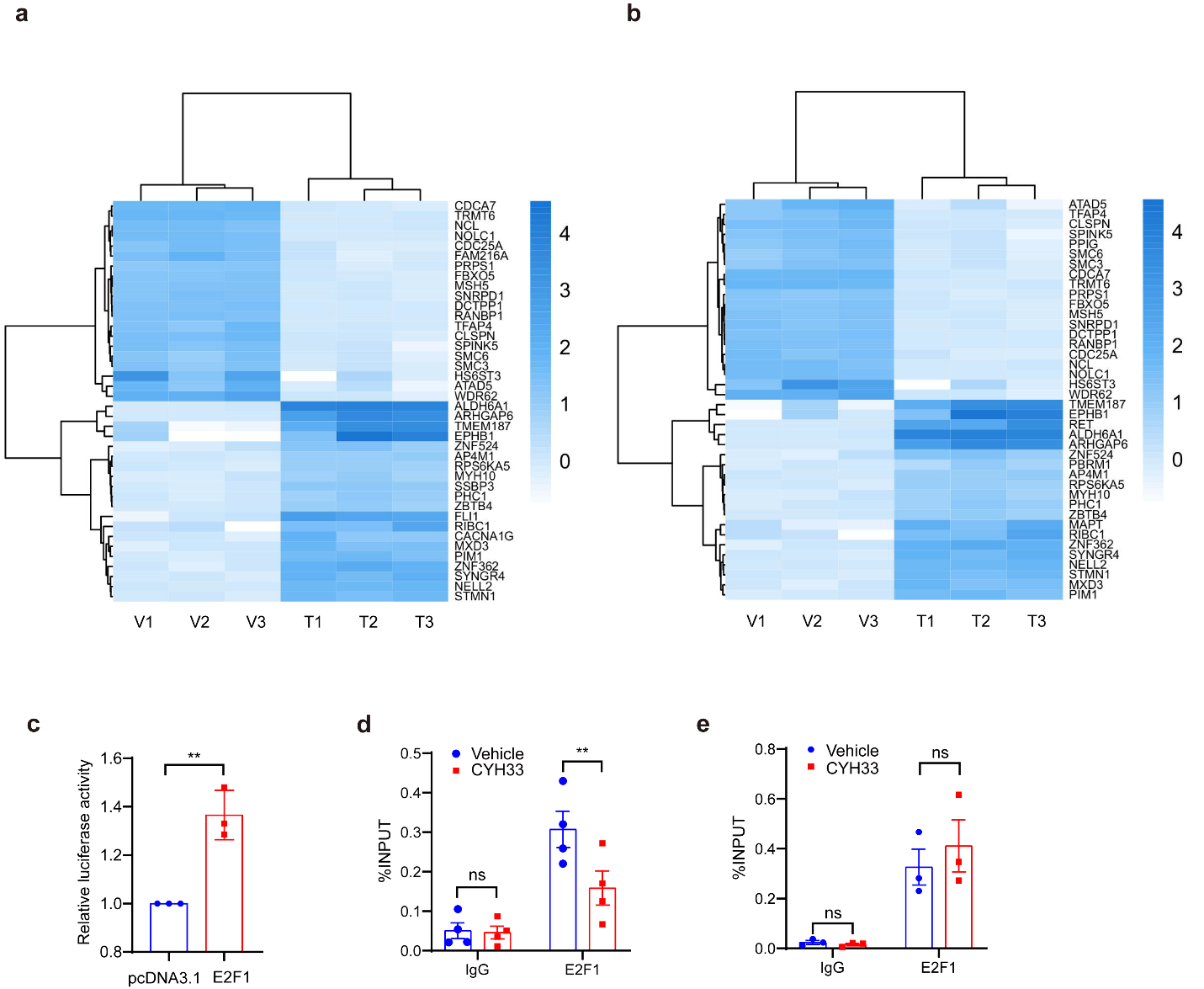


Figure. S6. CYH33 down-regulated the expression of SKP2 via attenuating E2F1 mediated transcription.

(a-b) Top 20 up-regulated and down-regulated genes in the gene sets of E2F1_Q6 (a) and E2F_02 (b) were shown in heatmaps. (c) 1 μg of SKP2-pGL4.10 plasmid and 1 μg of control vector or E2F1-expressing plasmid were co-transfected with 200 ng of pGL4.74 vector into HEK293T cells. Luciferase activity was measured 48 h after the transfection. p value was determined by two-tailed unpaired Student's t-test. **, p < 0.01. (d-e) ChIP assays were performed with the antibody against E2F1 in KYSE410 (d, n = 4) or KYSE450 (e, n = 3) cells treated with CYH33 (1 μM) for 24 h. Fold enrichment of E2F1 in the promoter region of *CDC6* was presented*.* Data were presented as mean ± SEM. p values were analyzed using two-tailed one-way ANOVA with Tukey multiple group comparison test. ns, p > 0.05; **, p < 0.01.


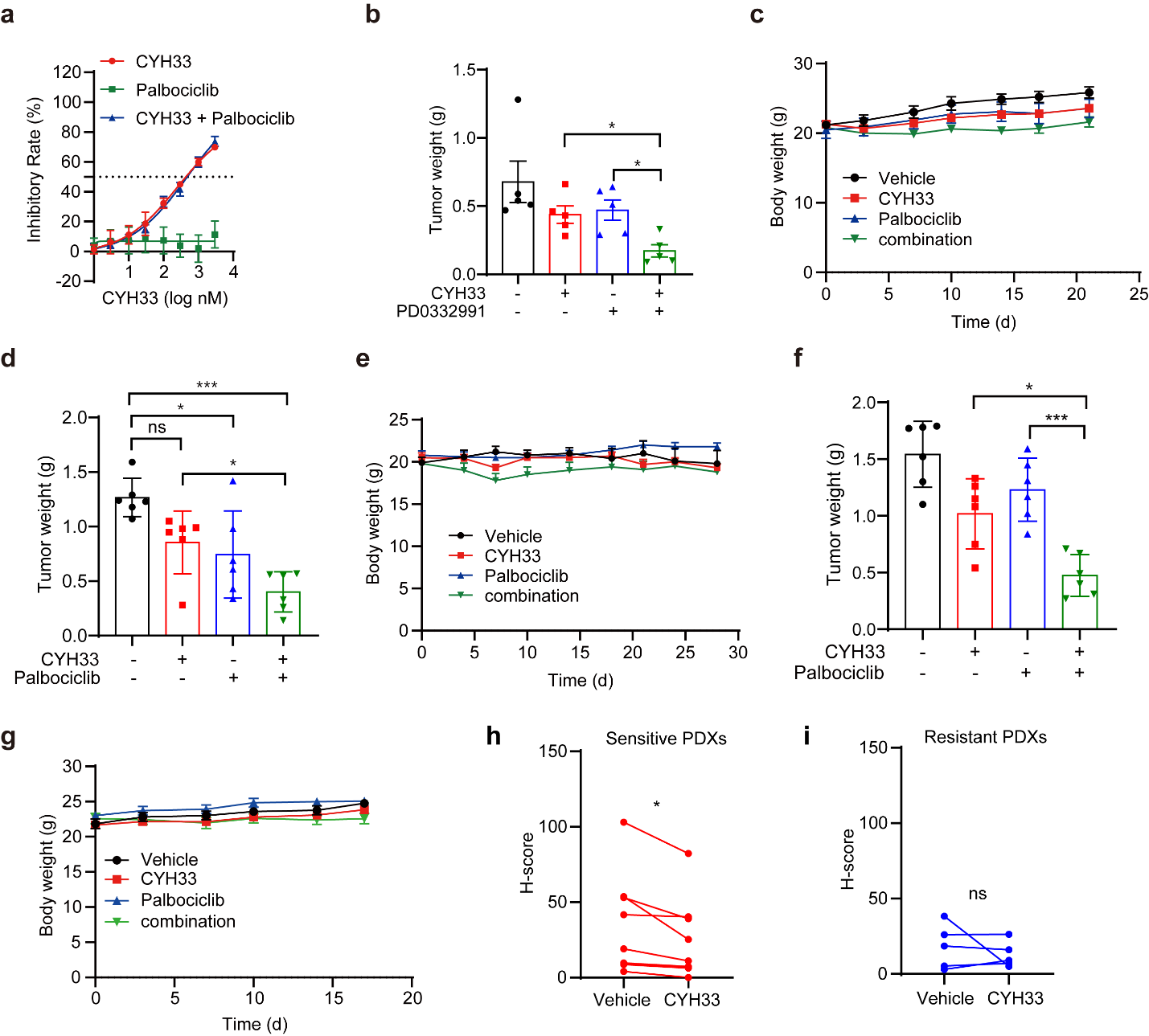


Figure. S7. CDK4/6 inhibitors sensitized ESCC to CYH33.

(a) TE-1 cells were treated with CYH33 and palbociclib alone or concurrently for 72 h. Cell proliferation was measured by SRB assay and CI values were determined by CalcuSyn software (n=3). (b-c) Randomly grouped BALB/c nude mice bearing KYSE450 xenografts were administrated orally with a vehicle control, CYH33 (10 mg/kg), palbociclib (50 mg/kg), or a combination of CYH33 and palbociclib once a day for 21 days (n = 5). (b) Tumor weight was measured at the end of the experiment. Data were presented as mean ± SEM. Differences between the indicated groups were analyzed using two-tailed one-way ANOVA with Tukey multiple group comparison test. *, p < 0.05. (c) Body weight was measured twice a week. (d-e) Randomly grouped BALB/c nude mice bearing EC040 xenografts were administrated orally with a vehicle control, CYH33 (10 mg/kg), palbociclib (50 mg/kg), or a combination of CYH33 and palbociclib once a day for 28 days (n = 6). (d) Tumor weight was measured at the end of the experiment. Data were presented as mean ± SEM. Differences between the indicated groups were analyzed using two-tailed one-way ANOVA with Tukey multiple group comparison test. ns, p > 0.05; *, p < 0.05; ***, p < 0.001. (e) Body weight was measured twice a week. (f-g) Randomly grouped BALB/c nude mice bearing EC036 xenografts were administrated orally with a vehicle control, CYH33 (10 mg/kg), palbociclib (50 mg/kg), or a combination of CYH33 and palbociclib once a day for 17 days (n = 6). (f) Tumor weight was measured at the end of the experiment. Data were presented as mean ± SEM. Differences between the indicated groups were analyzed using two-tailed one-way ANOVA with Tukey multiple group comparison test. *, p < 0.05; ***, p < 0.001. (g) Body weight was measured twice a week. (h-i) Expression level of cyclin D1 was measured in sensitive (h) and resistant (i) ESCC PDXs collected at the end of treatment by tissue microarray. Difference between groups was analyzed by two-tailed paired Student’s t-test. ns, p > 0.05; *, p < 0.05.
